# Veno-Venous Extracorporeal Membrane Oxygenation as Rescue Therapy for Near-Fatal Asthma: A Case Report

**DOI:** 10.7759/cureus.107865

**Published:** 2026-04-28

**Authors:** Mirza Muflihul Haque, Shahzadi Sayeeda Tun Nessa, Shihan Mahmud R Huq, Raziuddin Ahmed, Raihan Rabbani, Mufizul Islam Polash

**Affiliations:** 1 Department of Critical Care Medicine, Square Hospitals Limited, Dhaka, BGD; 2 Department of Critical Care Medicine, Hi Care Hospital, Dhaka, BGD

**Keywords:** acute severe asthma, invasive mechanical ventilation, near fatal asthma, pneumomediastinum, pulmonary barotrauma, refractory hypercapnia, status asthmaticus, veno-venous extracorporeal membrane oxygenation (vv ecmo)

## Abstract

Near-fatal asthma is a life-threatening condition characterized by severe airflow obstruction, refractory hypercapnia, and respiratory acidosis. Although invasive mechanical ventilation is often required, it carries significant risks, including barotrauma, dynamic hyperinflation, and hemodynamic compromise. In selected cases, extracorporeal membrane oxygenation (ECMO) can be used as a rescue therapy when conventional management fails. We report the case of a 19-year-old woman with near-fatal asthma who developed refractory respiratory acidosis and severe barotrauma despite maximal medical therapy and invasive mechanical ventilation. She presented with profound hypoxemia, with an oxygen saturation of 64% on room air. Due to persistent acidosis and hemodynamic instability, veno-venous ECMO was initiated as a salvage therapy. Immediately before ECMO initiation, arterial blood gas analysis showed severe acidosis with pH 7.00, partial pressure of carbon dioxide (PaCO2) 115 mmHg, and lactate 14 mmol/L. Within 12 hours of ECMO initiation, pH improved to 7.30, PaCO2 decreased to 49 mmHg, and lactate fell to 2.0 mmol/L, with concurrent hemodynamic stabilization. The patient showed marked clinical improvement and was successfully weaned from ECMO after four days and later liberated from mechanical ventilation following tracheostomy.

This case highlights VV-ECMO as a viable rescue strategy in carefully selected patients with refractory near-fatal asthma when conventional ventilation fails or risks further ventilator-induced lung injury.

## Introduction

Near-fatal asthma represents the most severe form of asthma exacerbation and is associated with severe airflow obstruction, dynamic lung hyperinflation, hypoxemia, and refractory hypercapnia. Approximately 2-4% of hospitalized patients with acute asthma require invasive mechanical ventilation, and mortality among ventilated patients remains significant [1,2]. While mechanical ventilation can be lifesaving, it may exacerbate lung injury through barotrauma, volutrauma, and hemodynamic compromise, especially in patients with severe bronchospasm. Extracorporeal membrane oxygenation (ECMO) provides extracorporeal gas exchange, allowing lung-protective ventilation and facilitating recovery while minimizing ventilator-induced lung injury. Several studies have demonstrated better survival outcomes in patients with near-fatal asthma supported with ECMO compared to other indications for extracorporeal life support [3]. However, the published evidence remains limited largely to registry analyses, reviews, and case-based reports, and real-world reports from resource-limited settings remain scarce. In addition, one of the most important practical questions is not simply whether ECMO can be used in near-fatal asthma, but when clinicians should recognize failure of conventional ventilation and escalate before further ventilator-induced injury occurs. We present a case of near-fatal asthma successfully managed with veno-venous ECMO after failure of maximal conventional therapy. This case highlights the clinical decision-making behind timely ECMO initiation in the setting of worsening hypercapnic acidosis and barotrauma despite optimized conventional management.

## Case presentation

**Figure 1:**
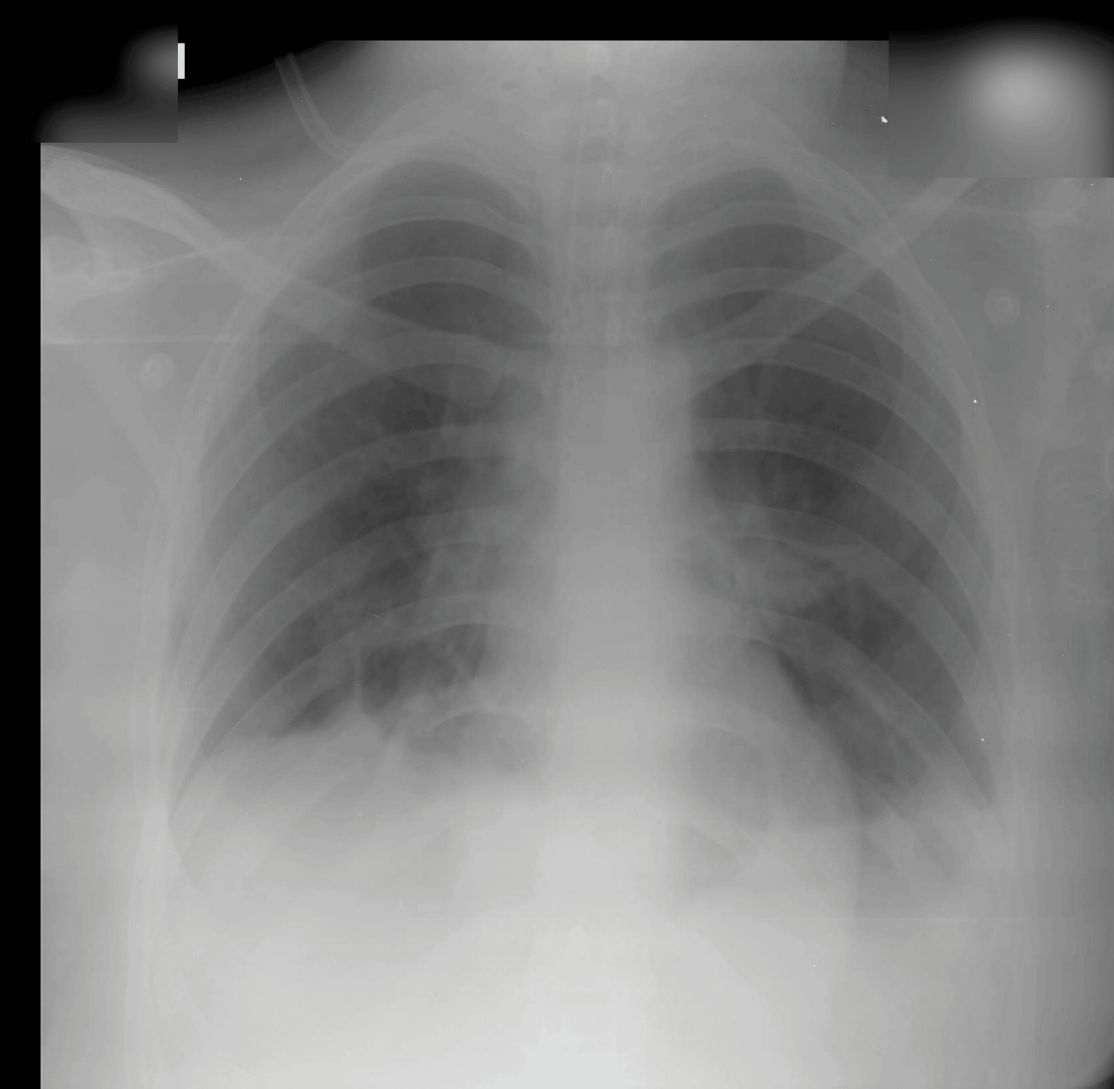
Initial portable chest radiograph on admission showing diffuse bilateral air-space opacities without pneumothorax

A 19-year-old woman, a known case of bronchial asthma, presented to the emergency department with sudden-onset severe shortness of breath following seafood ingestion. She had no prior hospital admissions for asthma and was not on regular controller inhaler therapy. On presentation, the patient was in severe respiratory distress. She was dyspneic, tachypneic, tachycardic, and cyanosed. Oxygen saturation was 64% on room air. Blood pressure was 120/70 mmHg. Chest examination revealed bilateral diffuse wheezing with markedly reduced air entry. Initial arterial blood gas (ABG) analysis demonstrated acute hypercapnic respiratory failure with respiratory acidosis (pH 7.23, partial pressure of carbon dioxide (PaCO2) 68 mmHg, partial pressure of oxygen (PaO2) 50 mmHg, HCO3- 28 mEq/L, lactate 3.0 mmol/L). Chest radiography showed diffuse bilateral air-space opacities without evidence of focal consolidation or pneumothorax (Figure 1).

The patient was treated aggressively with repeated nebulized salbutamol and ipratropium bromide, high-dose intravenous corticosteroids, and intravenous magnesium sulfate. Despite maximal medical therapy, her respiratory status continued to deteriorate with worsening hypoxemia and persistent respiratory acidosis. She was subsequently intubated under deep sedation and neuromuscular blockade and initiated on invasive mechanical ventilation. Ventilator settings included assist-control pressure control mode with a fraction of inspired oxygen (FiO2) of 1.0, respiratory rate of 14 breaths per minute, and inspiratory pressure of 30 cm H2O. Post-intubation ABG remained severely abnormal (pH 7.14, PaCO2 61 mmHg, PaO2 91 mmHg, HCO3- 20 mEq/L, lactate 7.0 mmol/L), indicating persistent respiratory and metabolic stress despite invasive ventilatory support.

**Figure 2:**
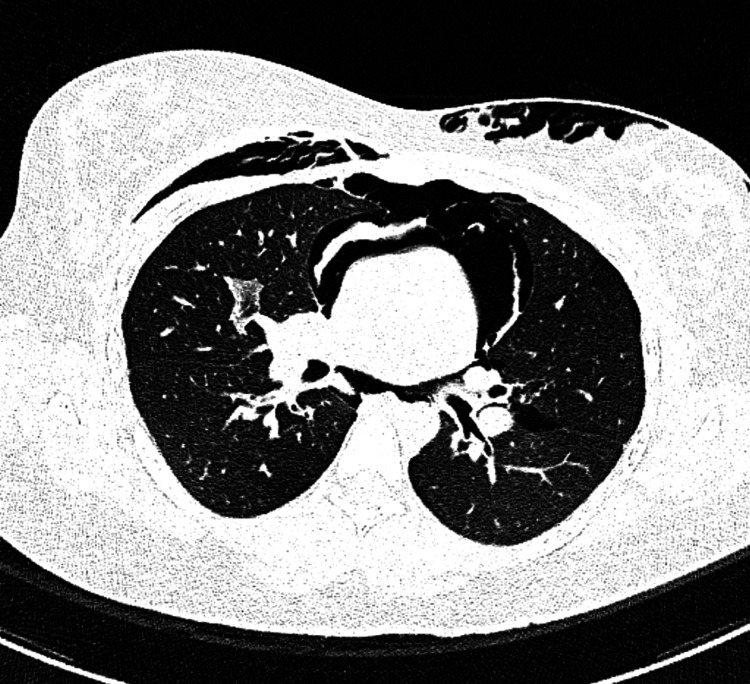
High-resolution computed tomography of the chest showing pneumomediastinum and extensive subcutaneous emphysema

During the course of mechanical ventilation, the patient developed extensive subcutaneous emphysema and pneumomediastinum due to severe dynamic lung hyperinflation (Figure 2).

Repeat ABG immediately prior to ECMO showed profound worsening, with pH 7.00, PaCO2 115 mmHg, PaO2 87 mmHg, HCO3- 34 mEq/L, and lactate 14 mmol/L. These findings indicated refractory hypercapnic respiratory failure with life-threatening acidosis despite maximal medical and ventilatory therapy. At this stage, further escalation of ventilatory pressures was considered unlikely to improve gas exchange and likely to worsen barotrauma. Given the failure of conventional therapy and ongoing life-threatening acidosis, a decision was made to initiate veno-venous ECMO as a rescue strategy.

**Figure 3:**
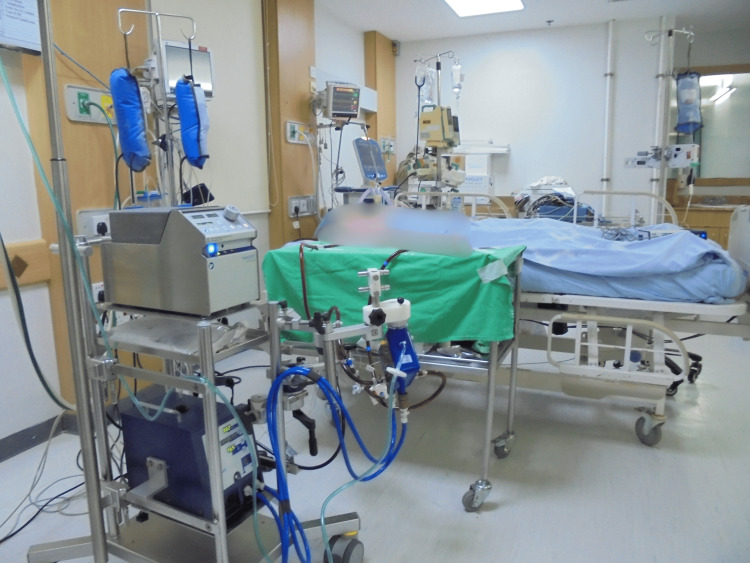
Patient supported with veno-venous extracorporeal membrane oxygenation following refractory hypercapnic respiratory failure

Veno-venous ECMO was established via cannulation of the right femoral vein and right internal jugular vein (Figure 3).

Systemic anticoagulation was maintained with heparin infusion. Following ECMO initiation, there was rapid correction of hypercapnia and acidosis (Table 1), demonstrating effective extracorporeal carbon dioxide removal and reversal of severe acid-base derangement. After 12 hours of ECMO support, ABG improved to pH 7.30, PaCO2 to 49 mmHg, PaO2 to 84 mmHg, HCO3- to 24 mEq/L, and lactate to 2.0 mmol/L. This physiologic improvement was accompanied by progressive hemodynamic stabilization and reduction in inotropic requirements.

**Table 1:** Serial arterial blood gas parameters before and after initiation of veno-venous extracorporeal membrane oxygenation ECMO: extracorporeal membrane oxygenation; FiO₂: fraction of inspired oxygen; PaCO₂: partial pressure of arterial carbon dioxide; PaO₂: partial pressure of arterial oxygen; HCO₃⁻: bicarbonate

Time point	Oxygen demand (FiO2)	pH ( _ )	PaCO2 (mmHg)	PaO2 (mmHg)	HCO3- (mEq/L)	Lactate (mmol/L)
Emergency room (Room air)	0.21	7.23	68	50	28	3.0
Post intubation	1	7.14	61	91	20	7.0
Pre ECMO	1	7.00	115	87	34	14
After 12 hours of initiation of ECMO	0.21	7.30	49	84	24	2.0
Reference range	-	7.35–7.45	35–45	80–100	22–26	<2

The patient demonstrated significant clinical improvement following ECMO initiation. After four days of ECMO support, she was successfully decannulated. Mechanical ventilation was continued with lung-protective strategies, and a tracheostomy was performed to facilitate weaning. The patient was successfully liberated from mechanical ventilation by day eight of hospitalization. The tracheostomy tube was subsequently removed without complications. After a total hospital stay of 16 days, the patient was discharged home in stable condition. At the one-month follow-up, she remained asymptomatic with good asthma control.

## Discussion

Near-fatal asthma represents a small but critical subset of asthma exacerbations characterized by refractory hypercapnia and respiratory failure despite maximal medical therapy. A small proportion of hospitalized patients require invasive mechanical ventilation, and outcomes remain poor in this group [1,2]. Mechanical ventilation in severe asthma is particularly challenging due to dynamic hyperinflation and intrinsic positive end-expiratory pressure, which may lead to barotrauma, haemodynamic compromise, and worsening gas trapping [3]. Although permissive hypercapnia is commonly used, it may be insufficient in patients with severe airflow obstruction, resulting in persistent acidosis and life-threatening hypercapnia [3]. Thus, the clinical challenge is not only to ventilate the patient, but to do so without worsening ventilator-induced lung injury.

Evidence supporting ECMO in near-fatal asthma has been increasingly reported in observational studies. Registry data have demonstrated favorable survival outcomes in asthma patients receiving ECMO support [4]. In this setting, veno-venous ECMO allows effective extracorporeal carbon dioxide removal while reducing ventilatory pressures and tidal volumes [5]. This facilitates lung-protective ventilation while reducing ventilator-induced lung injury.

Recent multicenter data have further supported the use of ECMO in refractory asthma with respiratory failure [6]. Additional case reports have also supported ECMO as an effective rescue therapy in refractory cases [7,8]. A recent case series by Gayen et al. described favorable clinical outcomes with rapid correction of hypercapnia following ECMO initiation [9]. A comprehensive review by Lozano-Espinosa et al. summarized the expanding evidence base for ECMO in near-fatal asthma [10]. More recently, Ibarra et al. reported further clinical experience with venovenous ECMO in near-fatal asthma [11]. A narrative review by Ekechukwu et al. also discussed outcomes of extracorporeal life support in acute severe asthma [12]. Additional contemporary case-based experience has been reported by Tan and Thornton [13]. Taken together, these reports support ECMO as a feasible rescue strategy in selected patients, but they also highlight that the most important practical question is identifying the point at which conventional ventilation is failing, and further escalation becomes more harmful than beneficial.

In the present case, ECMO was initiated due to persistent severe hypercapnia (PaCO2 115 mmHg) and worsening acidosis (pH 7.00) despite optimized mechanical ventilation. The patient had also developed pneumomediastinum and extensive subcutaneous emphysema, indicating that continued conventional ventilation was not only ineffective but also injurious. From a clinical standpoint, the inability to safely ventilate the patient without worsening barotrauma was a key factor in the decision to initiate ECMO.

The timing of ECMO initiation appears to be an important determinant of outcome. Early escalation, before the development of severe complications such as extensive barotrauma or prolonged exposure to high ventilatory pressures, may improve clinical outcomes, as suggested in observational studies [12]. In our patient, rapid improvement in pH, PaCO2, and lactate within 12 hours of ECMO initiation, followed by decannulation after 4 days and liberation from mechanical ventilation by day 8, supports the potential value of timely escalation once conventional ventilatory strategies are clearly failing.

Despite its benefits, ECMO remains associated with potential complications, including bleeding, thrombosis, infection, and vascular injury [13]. Therefore, careful patient selection is essential, particularly in patients with refractory hypercapnia and failure of conventional ventilation strategies [10,12]. Overall, available evidence supports ECMO as a life-saving rescue therapy in selected patients with near-fatal asthma, particularly when applied in a timely manner.

## Conclusions

This case demonstrates that VV-ECMO can be an effective rescue strategy in refractory near-fatal asthma when conventional ventilation fails to provide adequate gas exchange or risks worsening barotrauma. In our patient, progressive hypercapnia, severe acidosis, rising lactate, and evolving ventilator-associated injury signaled a point at which further escalation of mechanical ventilation was unlikely to be beneficial. The favorable clinical course after ECMO supports the importance of early recognition of failing conventional ventilation and timely escalation in appropriate cases. However, VV-ECMO should be considered a selective, evidence-supported rescue therapy for carefully chosen patients with reversible disease and life-threatening ventilatory failure, rather than a routine intervention for severe asthma exacerbations.
